# Synthesis, Biological Evaluation and Molecular Docking of Certain Sulfones as Potential Nonazole Antifungal Agents

**DOI:** 10.3390/molecules21010114

**Published:** 2016-01-20

**Authors:** Mohamed Fares, Mohamed A. Said, Muhammad A. Alsherbiny, Radwa A. Eladwy, Hadia Almahli, Marwa M. Abdel-Aziz, Hazem A. Ghabbour, Wagdy M. Eldehna, Hatem A. Abdel-Aziz

**Affiliations:** 1 Department of Pharmaceutical Chemistry, Faculty of Pharmacy, Egyptian Russian University, Badr City, Cairo 11829, Egypt; madyyy_@hotmail.com (M.A.S.); hadia.almahli@yahoo.com (H.A.); wagdy2000@gmail.com (W.M.E.); 2 Pharmacognosy Department, Faculty of Pharmacy, Cairo University, Kasr El-Aini Street, Cairo 11562, Egypt; muhammad.alsherbiny@pharma.cu.edu.eg; 3 Department of Pharmacology and Toxicology, Faculty of Pharmacy, Egyptian Russian University, Badr City, Cairo 11829, Egypt; radwa.eladwy22@gmail.com; 4 The Regional Center for Mycology and Biotechnology, Al-Azhar University, Cairo 11759, Egypt; marwa2rcmb@yahoo.com; 5 Department of Pharmaceutical Chemistry, College of Pharmacy, King Saud University, P. O. Box 2457, Riyadh 11451, Saudi Arabia; ghabbourh@yahoo.com; 6 Department of Medicinal Chemistry, Faculty of Pharmacy, Mansoura University, Mansoura 35516, Egypt; 7 Department of Applied Organic Chemistry, National Research Center, Dokki, Giza 12622, Egypt

**Keywords:** synthesis, sulfone, antifungal, anticandidal, docking

## Abstract

We reported herein the synthesis, antifungal activity, docking and *in silico* ADME prediction studies of four novel series of sulfones **6a**–**f**, **8a**–**c**, **10a**–**f** and **12a**–**c**. All the newly synthesized sulfones were tested against four strains of *Candida* (including fluconazole-resistant *Candida*), two strains of *Aspergillus*, two dermatophytic fungi (*Trichophytons mentagrophyte* and *Microsporum canis*) and *Syncephalastrum* sp. with fluconazole as a reference drug. In general, compounds **8a** and **10b** showed selective and potent anticandidal activity (MIC: 0.19–0.81 µM) relative to fluconazole (MIC = 1.00 µM). Furthermore, **10e** and **12a** elicited a remarkable and selective antifungal activity against *Aspergillus* sp. and the dermatophytic fungi (MIC: 0.16–0.79 µM) relative to fluconazole (MIC: 2–2.6 µM). Moreover, the docking results of the sulfones **6a**, **8a**, **10a** and **10b** at the active site of CYT P450 14α-sterol demethylase showed a comparable binding interaction (interaction Energy = −34.87 to −42.43 kcal/mol) with that of fluconazole (IE = −40.37 kcal/mol).

## 1. Introduction

In recent decades, fungal infection is considered as an escalating public health problem and causes a continuous and serious threat to humans and animals [1,2,3]. The increasing incidence of fungal infections is mainly due to evolution of the resistance to antifungal agents and the growing number of immunocompromised patients [4,5]. In addition, mutation of the fungal species and the selection pressure caused by the overuse of antifungal agents in humans and animals over the past 75 years are considered the most crucial reasons of resistance [6]. Moreover, the spread of antifungals’ resistance all over the world arises as a consequence of inter species gene transmission, poor sanitation conditions and the growing prevalence of nosocomial infection [6].

Invasive fungal infections (IFIs) and dermatomycoses have increased significantly since the second half of the 20th century mainly because of the large number of individuals with higher vulnerability such as neonates, cancer patients, those facing long hospital stays or infected with acquired immunodeficiency virus [7,8]. Surveillance data indicated that there are fundamentally three opportunistic species responsible for the IFIs; *Candida albicans*, *Cryptococcus* *neoformans* and *Aspergillus fumigatus*. Furthermore, the epidemiology of IFIs has shifted towards non-*albicans Candida*, non-*fumigatus Aspergillus* [7], this shift caused by the use of millions of tons of the antifungal prophylaxis therapy. Regarding the candidiasis, *C. albicans* was the most common species, accounting for about more than half of all cases (54%) followed by *C. glabrata* (19%), *C. parapsilosis* (11%), *C. tropicalis* (11%), and *C. krusei* (2%) [9]. Generally, one fifth of the population at any given time are infected with dermatophytes, which are fungi causing localized infection of the hair, nail and stratum corneum. *Trichophyton*, *Microsporum* and *Epidermophyton* are the most abundant genera of dermatophytes that account for the majority of dermatomycoses [10,11].

**Figure 1:**
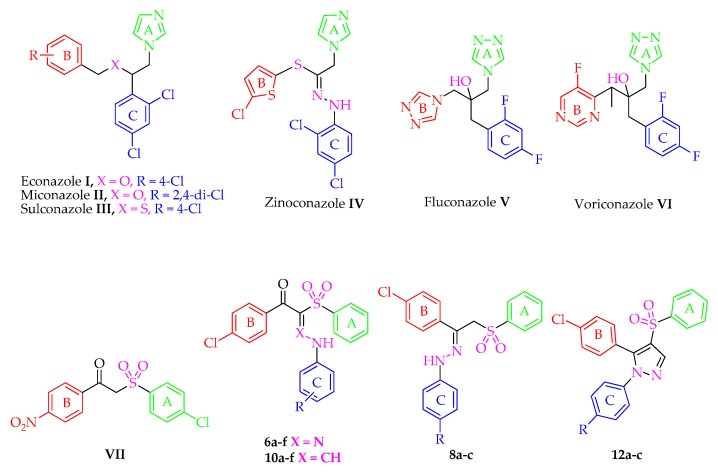
Structures of antifungal drugs **I**–**VII** and the novel entitled compounds **6a**–**f**, **8a**–**c**, **10a**–**f** and **12a**–**c**.

Unfortunately, there is a limited arsenal of the antifungal chemotherapies including polyenes like amphotericin B and azoles such as voriconazole and fluconazole [12,13]. Furthermore, these approved drugs may present some undesirable side effects such as drug interaction, bioavailability and hepatotoxicity [14,15]. Azole antifungals **I**–**VI** (Figure 1) exert their effect via the inhibition of cytochrome P450 dependent enzyme lanosterol 14α-demethylase (Cyp 51). Cyp 51 is an important enzyme required for the synthesis of ergosterol from its precursor lanosterol. Ergosterol is a vital component of the fungal cell membrane [16]. In addition, with extensive use and prolonged therapy of the antimycotic azoles on different *Candida* species cells, the fungal cells adapt to be eventually azole resistant [17].

Fluconazole **V**, the first member of this class, demonstrates an excellent activity against most *Candida* species but has less activity against *C. glabrata* and no apparent activity against *C. krusei* [18]. Fluconazole **V** and voriconazole **VI** are the drugs of choice for the treatment of severe systemic mycoses and invasive aspergillosis, respectively [19,20]. On the other hand, because of the similarity between mammalian and fungal cells in the composition of cell membrane, azoles are not entirely selective to fungi [12]. They are reported to be hepatotoxic due to the fact that they undergo hepatic metabolism. Consequently, it is recommended that patients who are administering any of this class should be carefully monitored for hepatotoxicity [16,21]. Although the azole ring is the most important pharmacophore for the antimycotic activity of this class, it is responsible too for the undesirable hepatotoxicity side effect [19]. Therefore, owing to the limited success of azoles, due to resistance and toxicity, there is urgent need to develop new antifungal agents that combine safety with antifungal efficacy against the most common fungi and against fluconazole-resistant strains.

Aryl sulfones are reported to possess strong antifungal activity [22,23,24,25]. Curti *et al.* [22] proved that the aryl sulfone derivative **VII** exhibited antifungal activity against *Candida tropicalis*. Further investigation on this interesting scaffold was done by our research group and resulted in the discovery of the broad spectrum anticandidal agent **8a** (R = H) [25]. In the present work, it was thought worthwhile to extend and optimize our previous investigation regarding the antifungal activity of the phenyl sulfone derivatives via modifications of the linker type and substitution of ring C of **8a** (R = H) with the prime aim to obtain a more potent and safe antifungal agent especially against drug resistant strains.

In view of the facts mentioned above and as a part of our ongoing effort to prepare anti-microbial agents [26,27,28], herein we present the design and synthesis of a focused library of novel aryl sulfone analogs **6a**–**f**, **8a**–**c**, **10a**–**f** and **12a**–**c** and evaluation of their anti-fungal activity against different invasive fungi (fluconazole-resistant *Candida albicans*, *Candida albicans*, *Candida tropicalis*, *Candida parapsilosis*; *Aspergillus fumigatus* and *Aspergillus niger*) and dermatophytic Fungi (*Trichophytons mentagrophytes* and *Microsporum canis*).

## 2. Results and Discussion

### 2.1. Chemistry

**Scheme 1:**
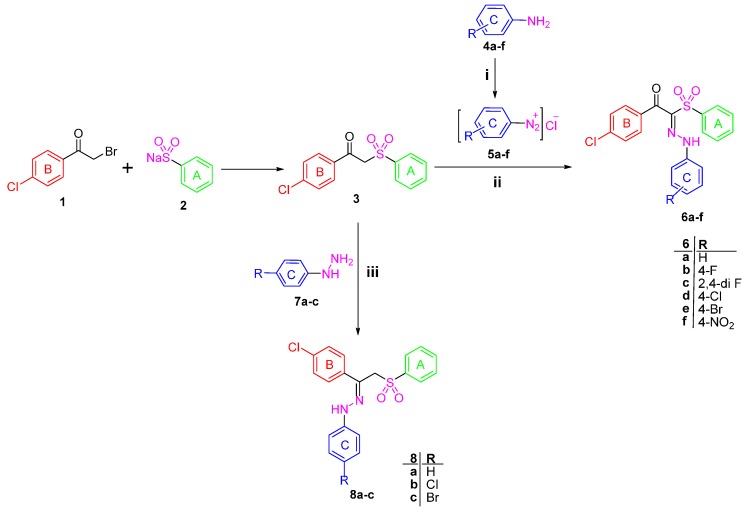
Synthesis of Target compounds **6a**–**f** and **8a**–**c**. *Reagents and conditions*: (**i**) NaNO_2_/HCl/0–5 °C; (**ii**) CH_3_COONa/EtOH/0–5 °C; (**iii**) EtOH/Catalytic amount of glacial acetic acid/reflux 6 h.

**Scheme 2:**
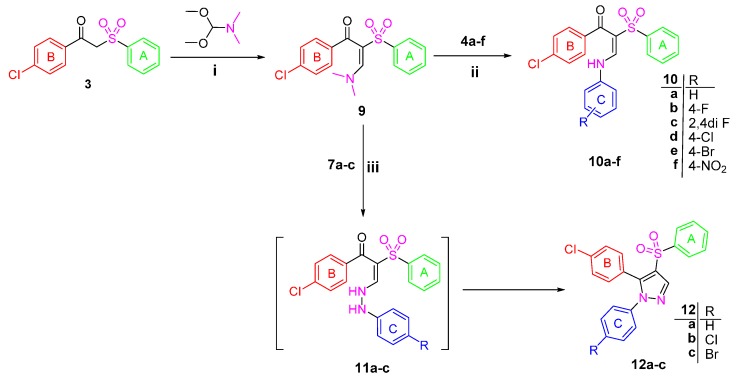
Synthesis of Target compounds **10a**–**f** and **12a**–**c** *Reagents and conditions*: (**i**) Neat reaction/reflux 4 h; (**ii**) Glacial acetic acid/reflux 2 h; (**iii**) EtOH/reflux 6 h.

**Figure 2:**
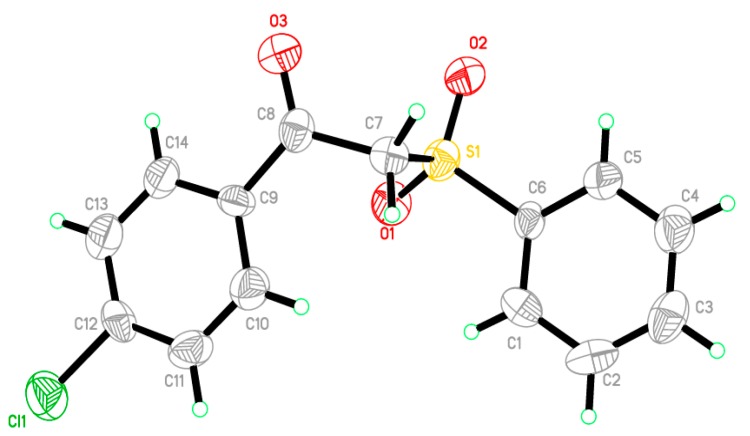
An ORTEP diagram of final X-ray structure of compound **3**.

1-(4-Chlorophenyl)-2-(phenylsulfonyl)ethanone **3** [22] was used as a starting material to obtain all the new targeted sulfone derivatives (Scheme 1 and Scheme 2). Furthermore, X-ray single crystal analysis for compound **3** gave an absolute confirmation for the structure. CCDC 1430111 contains the supplementary crystallographic data for this paper (see Tables S1–S3 for details) (Figure 2). These data can be obtained free of charge via http://www.crystallography.net/search.html. Diazotization of aromatic amines **4a**–**f** with hydrochloric acid and sodium nitrite afforded the diazonium salts **5a**–**f** which subsequently coupled with the active methylene of the phenyl sulfone derivative **3** in the presence of sodium acetate trihydrate and ethanol to afford the corresponding hydrazones **6a**–**f** [29].

^1^H-NMR spectra of the hydrazone derivatives **6a**–**f** showed D_2_O exchangeable singlet signals in the regions δ 11.46–11.77 and 12.30–12.44 ppm attributable to the resonating NH group, in addition to the disappearance of the singlet signal of methylene group of **3**. The phenyl sulfone **3** was further reacted with phenylhydrazine derivatives **7a**–**c** in ethanol at ambient temperature to afford the title compounds **8a**–**c** (Scheme 1). The ^1^H-NMR of the hydrazones **8b** and **8c** exhibited the exchangeable signal of the NH group which appeared at δ 9.86 and 9.87 ppm and the signal of CH_2_ protons appeared as singlet at δ 5.14 and 5.15 ppm, whereas the ^13^C-NMR of **8c** revealed the signal of CH_2_ carbon at δ 70.30 ppm.

In order to obtain more structural variations of the main phenyl sulfone scaffold to explore and understand more about the structure activity relationship, the starting sulfone **3** was reacted with the highly reactive *N*,*N*-dimethylformamide dimethyl acetal (DMFDMA) to obtain 1-(4-chlorophenyl)-3-(dimethylamino)-2-(phenylsulfonyl)prop-2-en-1-one **9** [30]. The structure of the enaminone derivative **9** was confirmed using different spectroscopic techniques. ^1^H-NMR revealed the disappearance of the methylene group singlet signal and the presence of the methine group signal at δ 8.05 ppm, while the two methyl groups protons appeared at δ 3.09 ppm. Moreover, ^13^C-NMR of **9** showed the signal of the methine sp^2^ carbon at δ 105.49 ppm and the signal of the sp^3^ methyl groups at δ 41.73 ppm.

The enaminone derivative **9** reacted with aniline derivatives **4a**–**f** and afforded 1-(4-chlorophenyl)-3-(arylamino)-2-(phenylsulfonyl)prop-2-en-1-ones **10a**–**f**. The latter phenyl sulfone derivatives are assumed to be formed by the nucleophilic substitution of the anilines **4a**–**f** on the activated double bond in the enaminones **9** followed by elimination of the dimethylamine.

The structures of the reaction products were assigned based on their elemental analyses and spectral data. Moreover, the ^1^H-NMR spectrum of the compounds **10a**–**f** revealed the presence of the two doublet signals of the two configurations of enaminone (CH = N signal) at δ 7.88–8.07 and 8.02–8.55 ppm. Interestingly, upon deuteration, the doublet signals disappeared in line with the appearance of singlet peak. Similarly, the exchangeable peak of the NH group appeared as doublet, while, upon adding D_2_O it is disappeared completely.

The synthesis of the pyrazole derivatives **12a**–**c** was achieved via the reaction of compound **9** with phenyl hydrazine derivatives **7a**–**c**. Furthermore, the compounds **12a**–**c** showed characteristic ^1^H-NMR signals in the region δ 8.34–8.37 ppm corresponding to the pyrazole proton and disappearance of the methine and methyl protons signals that are present in the enamine derivative **9** [31].

### 2.2. Biological Data

#### 2.2.1. *In Vitro* Antifungal Activities

The antifungal activity was performed at The Regional Center for Mycology and Biotechnology (RCMB), Al-Azhar University, Cairo, Egypt.

As depicted in Table 1 and Table 2, target compounds **6a**–**f**, **8a**–**c**, **9**, **10a**–**f**, **12a**–**c** and fluconazole, as antifungal reference drug, were evaluated *in vitro* for their antifungal activity by inhibition zone technique and minimum inhibitory concentration (MIC) against fluconazole-resistant *Candida albicans* (RCMB 05036, *Ca^R^*), *Candida albicans* (RCMB 05079, *Ca*), *Candida tropicalis* (RCMB 05142, *Ct*), *Candida parapsilosis* (RCMB 05324, *Cp*), *Aspergillus fumigatus* (RCMB 02568, *Af*), *Aspergillus niger* (RCMB 02542, *An*), *Trichophytons mentagrophyte* (RCMB 09285, *Tm*), *Microsporum canis* (RCMB 09321, *Mc*) and *Syncephalastrum racemosum* (RCMB 05922). All the compounds showed no activity against *Syncephalastrum racemosum*. However, data in Table 1 and Table 2 revealed that the unsubstituted hydrazone and unsubstituted aniline phenyl sulfone counterparts **6a** and **10a,** respectively, showed significant broad antifungal activity against most of the tested species.

Observing the results, we could deduce some valuable data about the structure activity correlation of the tested phenyl sulfone derivatives. Firstly, we explored that the substitution of ring C has an impact of the antimycotic spectrum and extent of inhibition. For example, among all the first series compounds **6a**–**f**, incorporation of α-unsubstituted phenyl hydrazone group led to compound **6a** with broad and good activity against all the invasive and dermatophytic fungi (MIC = 1.57–3.14 µM) relative to fluconazole (MIC = 1.00–2.60 µM). However, substitution with any other group abolished the activity, which may be attributed to solubility problems. Similarly, bioisosteric replacement of N of the hydrazone group with CH in **6a** led to **10a** counterpart with broad spectrum activity against most of the tested compounds (MIC = 1.57–3.14 µM). However, compound **10b**, bearing fluorine substituent at the 4-position, showed an excellent activity and selectivity against the tested *Candida* sp. (MIC = 0.19 µM) relative to fluconazole (no activity against *Ca^R^* and MIC = 1 µM against *Ca*, *Ct* and *Cp*). Furthermore, grafting bromine, compound **10e**, shifted the activity towards the *Aspergillus* sp. and dermatophytic fungi (MIC = 0.16–0.66 µM) relative to the reference drug (MIC = 2.00–2.60 µM). Conversely, substitution with bulky electron withdrawing groups such as 2,4-difluoro (**10c**), 4-Cl (**10d**) and 4-NO_2_ (**10f**) abolished the antifungal activity.

On the other hand, among the second series compounds, **8a**–**c**, the unsubstituted phenyl hydrazone derivative displayed selective and potent activity against all the *Candida* sp. (MIC = 0.20–0.81 µM) relative to fluconazole (no activity against *Ca^R^* and MIC = 1 µM against *Ca*, *Ct* and *Cp*). Further substitution with 4-chloro or 4-bromo resulted in loss of the activity. Rigidification of the hydrazone group of **8a**–**c** into pyrazole ring resulted in **12a**–**c** analogs. Incorporation of unsubstituted phenyl group to the pyrazole derivative, compound **12a**, showed a remarkable activity being 13-fold, 2.5-fold, 2.5-fold and 10-fold more active the fluconazole against *A. fumigatus*, *A. niger*, *T.* *mentagrophyte* and *M. Canis*, respectively. Substitution with 4-chloro **12b** or 4-bromo **12c** led to complete loss of activity, which again may be due to solubility problems.

Interestingly, we identified two new broad spectrum antifungal agents, **6a** and **10a**, while two phenyl sulfone counterparts, **8a** and **10b**, possessed strong inhibition of three *Candida* sp. (*C. albicans*, *C. tropicalis* and *C. parapsilosis*), three compounds, **6a**, **8a** and **10b**, were shown to fight against fluconazole-resistant *Candida albicans* and two novel agents had excellent anti-aspergillosis and anti-dermatophytic activity, **10e** and **12a**. However, further iterative cycles of optimization of ring A and ring B of the active compounds **6a**, **8a** and **10b** are ongoing in our lab for lead identification and development with strong activity against fluconazole-resistant species.

**Table 1:** Mean zone of inhibition in mm ± Standard deviation beyond well diameter (6 mm) produced on a range of environmental and clinically pathogenic microorganisms using (125 µg/mL) concentration of tested samples.
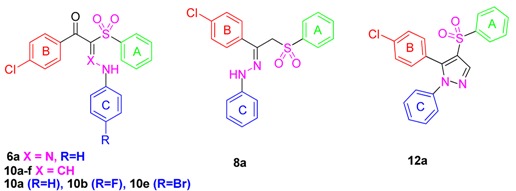

Comp.	Invasive Fungi						Dermatophytic Fungi	
	*Ca^R^*	*Ca*	*Ct*	*Cp*	*Af*	*An*	*Tm*	*Mc*
**6a**	14.2 ± 0.58	12.4 ± 0.63	11.5 ± 0.58	12.4 ± 0.63	11.2 ± 0.63	11.9 ± 0.63	9.3 ± 0.63	10.2 ± 0.63
**8a**	18.2 ± 0.72	20.3 ± 0.63	19.4 ± 0.58	20.6 ± 0.63	NA	NA	NA	NA
**10a**	NA	12.3 ± 0.63	14.1 ± 0.63	15.2 ± 0.58	10.6 ± 0.72	9.3 ± 0.58	10.1 ± 0.63	12.1 ± 0.58
**10b**	19.6 ± 0.63	20.9 ± 0.72	21.2 ± 0.45	22.4 ± 0.58	NA	NA	NA	NA
**10e**	NA	NA	NA	NA	20.3 ± 0.63	18.2 ± 0.63	17.1 ± 0.63	18.9 ± 0.63
**12a**	NA	NA	NA	NA	19.3 ± 0.72	18.1 ± 0.58	18.2 ± 0.72	20.1 ± 0.58
**Flu**	NA	16.6 ± 0.58	15.4 ± 0.58	17.2 ± 0.72	19.6 ± 0.58	16.3 ± 0.63	13.6 ± 0.72	15.4 ± 0.63

Only the compounds with I.Z > 9 mm are mentioned. NA: No Activity (I.Z < 9 mm). The screening organisms, Invasive Fungi: fluconazole resistant *Candida albicans* (RCMB 05036, *Ca^R^*), *Candida albicans* (RCMB 05079, *Ca*), *Candida tropicalis* (RCMB 05142, *Ct*), *Candida parapsilosis* (RCMB 05324, *Cp*); *Aspergillus fumigatus* (RCMB 02568, *Af*) and *Aspergillus niger* (RCMB 02542, *An*). Dermatophytic Fungi: *Trichophytons mentagrophytes* (RCMB 09285, *Tm*) and *Microsporum canis* (RCMB 09321, *Mc*); Flu: Fluconazole.

**Table 2:** Antimicrobial activity as MICs (µM) of fluconazole and synthesized compounds against tested microorganisms and selectivity index (SI) of the synthesized compounds.

Comp.	Invasive Fungi						Dermatophytic Fungi		PC3
	*Ca^R^*	*Ca*	*Ct*	*Cp*	*Af*	*An*	*Tm*	*Mc*	IC_50_
**6a**	1.57	1.57	1.57	1.57	1.57	1.57	3.14	1.57	38.86
	(24.75)	(24.75)	(24.75)	(24.75)	(24.75)	(24.75)	(12.38)	(24.75)	
**8a**	0.81	0.20	0.41	0.20	NA	NA	NA	NA	37.36
	(46.12)	(>100)	(91.12)	(>100)					
**10a**	NA	1.57	1.57	1.57	1.57	3.14	1.57	1.57	35.14
		(22.38)	(22.38)	(22.38)	(22.38)	(11.19)	(22.38)	(22.38)	
**10b**	0.19	0.19	0.19	0.19	NA	NA	NA	NA	37.09
	(>100)	(>100)	(>100)	(>100)					
**10e**	NA	NA	NA	NA	0.16	0.66	0.66	0.66	38.73
					(>100)	(58.68)	(58.68)	(58.68)	
**12a**	NA	NA	NA	NA	0.20	0.79	0.79	0.20	28.90
					(>100)	(36.6)	(36.60)	(>100)	
**Fluconazole**	NA	1	1	1	2.6	2	2	2	

#### 2.2.2. *In Vitro* Cytotoxicity

*In vitro* cytotoxicity of the most active compounds **6a**, **8a**, **10a**, **10b**, **10e** and **12a** was evaluated against human prostate cancer PC-3 cells using sulforhodamine B (SRB) colorimetric assay. The IC_50_ values obtained for these compounds are listed in Table 2. Moreover, selectivity index (SI) is used to estimate the therapeutic effect of a drug and to identify drug candidates for further studies. SI of each compound was determined as the ratio of IC_50_ to MIC (Table 2). It was reported that new drugs candidates must have SI equal or higher than 10 [32]. In this study, all the tested compounds could be considered as promising new antifungal drug candidates with SI > 11.19.

### 2.3. Computational Study

#### 2.3.1. Docking

**Figure 3:**
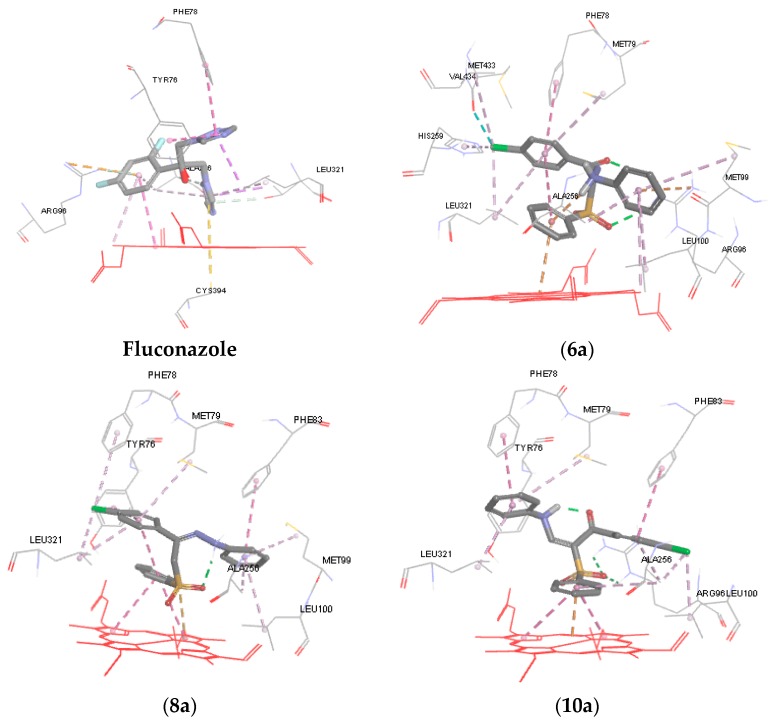

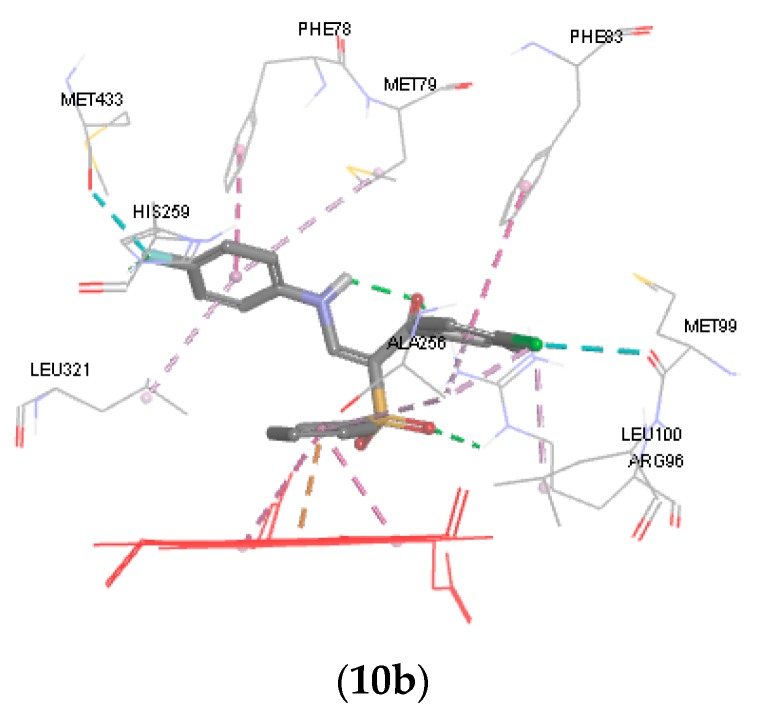
3D representations of the binding patterns of fluconazole, **6a**, **8a**, **10a** and **10b** into the binding site cytochrome P450 14α-sterol demethylase (PDB code 1EA1).

To further understand the mechanism of action of the synthesized compounds, molecular modeling and docking studies of **6a**, **8a**, **10a** and **10b** were performed on the X-ray crystal structure of cytochrome P450 14α-sterol demethylase from *Mycobacterium tuberculosis* (*Mycobacterium* P450 DM) and co-crystallized fluconazole (PDB code: 1EA1) using Discovery Studio 4/CDOCKER protocol (Accelrys Software Inc., San Diego, CA, USA*)*. It is well known that fluconazole exerts its antifungal activity by binding to Cyp-P450 DM enzyme. The nitrogen of azole ring of fluconazole coordinated to the heme iron, while the azole ring itself in the fluconazole structure is positioned almost perpendicular to the porphyrin plane (cofactor) [33]. Moreover, the 2,4-difluorophenyl forms a pi-cation interaction with Arg96 and the other triazole ring formed a pi-pi hydrophobic interaction with Tyr76 and Phe78 (Figure 3) [33].

In general, data obtained from docking shows that all the docked hits can interact with porphyrin plane and occupy the binding site of the enzyme with strong binding interaction energy ranging from −34.873 to −42.431 kcal/mol relative to fluconazole (−40.374 kcal/mol) (Table 3). As illustrated in Figure 3, in case of compounds **6a**, ring B forms a pi-cation interaction with the heme iron, while in compound **8a** the oxygen of sulfone moiety is perpendicular to the porphyrin plane heme ion. The p-chlorophenyl (ring A) of **6a** and **8a** forms a hydrophobic interaction (pi-pi interaction) with Phe78, Met79, and Leu321 and almost occupies the same hydrophobic pocket. Similarly, ring C of **6a** and **8a** extends into the unoccupied hydrophobic region above the heme ring and showed good van der Waals interactions with amino acids Met99, Leu100, Ala256. The carbonyl group of the hydrazone derivative **6a** and the sulfone group form a hydrogen bond with the side chain of Arg96, while the amino acid formed a pi-cation interaction with fluconazole.

**Table 3:** Docking scores of the most active compounds and fluconazole.

Compound	CDOCKER Interaction Energy (kcal/mol)
**6a**	−42.431
**8a**	−36.214
**10a**	−34.873
**10b**	−39.229
**Fluconazole**	−40.374

The docking result revealed that the phenyl sulfone group, the p-chlorophenyl group and the aniline side chain of **10a** and **10b** can bind in the same manner to the active site of CYP51. Ring B forms a pi-cation interaction with heme ion and pi-pi interaction with the pyrrole rings of porphrin. Ring A of **10a** and **10b** forms hydrophobic and van der Waals interactions with surrounding hydrophobic residues such as Phe83, Leu100, and Ala256. It was observed that the anilino group (ring C) of **10a** occupies the hydrophobic pocket above the heme ring and interacts with Phe78, Met79 and Leu321. The p-fluoro anilino group of **10b** affords more hydrophobic interaction points with the hydrophobic pocket and forms a hydrophobic interaction with Phe78, Met79, His259 Met433 and Leu321. Finally, the sulfone group forms a hydrogen bond with the side chain of Arg96.

**Figure 4:**
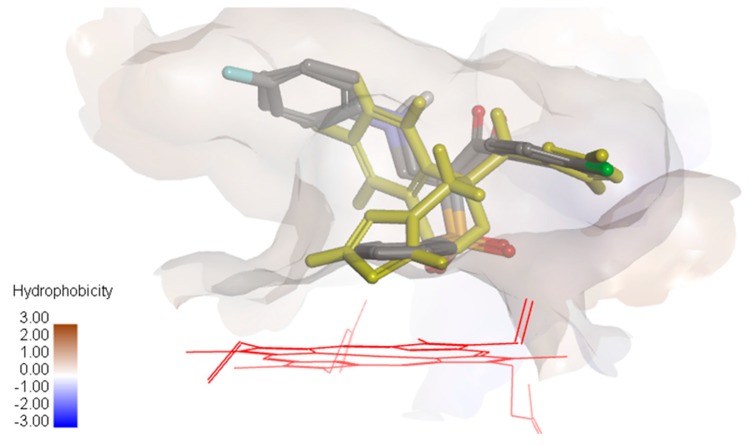
Overlay of compounds **10a** and **10b** and fluconazole (yellow) showing the hydrophobic surface in the groove of cytochrome P450 14α-sterol demethylase.

It was observed that compounds **10a** and **10b** were oriented in the binding groove of enzyme in such a fashion that favors the possibility of pi-pi interaction of the three benzene rings with the hydrophobic amino acid residues of the binding site of the enzyme (Figure 4). Overlaying the bioactive conformers of **10a** and **10b** on fluconazole showed that both the counterparts extend with the 4-chlorophenyl and anilino fragments into hydrophobic pockets above the porphyrin ring. Our future study will be directed towards optimizing the biology and modeling results in order to obtain a more active compound.

#### 2.3.2. ADME and Molecular Property Prediction

**Figure 5:**
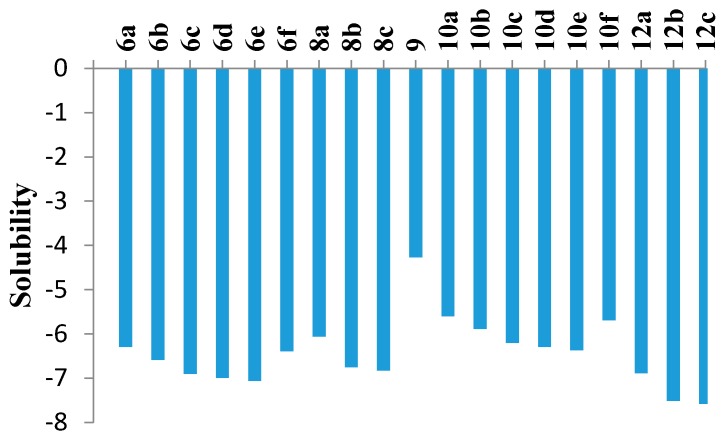
Computer aided solubility prediction of the sulfone derivatives (Solubility parameter. (0~−2 = optimal, −2~−4 = good, −4~−6 = low, −6~−8 = very low).

The ADME of the phenyl sulfone counterparts **6a**–**f**, **8a**–**c**, **9**, **10a**–**f** and **12a**–**c** was predicted via a theoretical kinetic study performed by means of the Discovery Studio software (Figure 5 and Table 4). Both AlogP98 and PSA (polar surface area) descriptors were calculated to evaluate the lipophilicity and polar surface area. Also, solubility, absorption and CYP2D inhibition levels were predicted. Most of the compounds showed extremely low solubility except **9**, **10a**, **10b** and **10f**. Finally, all members were predicted to be CYP2D non-inhibitors. It is noteworthy that careful study of the molecular properties suggested that the inactivity of most of the substituted derivatives may be due to the high lipophilicity and low solubility.

**Table 4:** Computer aided ADME and molecular property prediction of the sulfone derivatives.

Comp.	AlogP98 ^a^	PSA ^b^	Solubility Level ^c^	Absorption Level ^d^	CYP2D6 Probability ^e^	Num_H Bond Donor	Num_H Bond Acceptor	Molecular Weight	Rotatable Bonds
**6a**	5.21	76.04	1	0	0.02	1	5	398.86	6
**6b**	5.42	76.04	1	1	0	1	5	416.85	6
**6c**	5.63	76.04	1	1	0	1	5	434.84	6
**6d**	5.88	76.04	1	1	0.02	1	5	433.31	6
**6e**	5.96	76.04	1	1	0.02	1	5	477.76	6
**6f**	5.11	118.86	1	2	0	1	8	443.86	7
**8a**	5.14	58.74	1	0	0	1	4	384.88	6
**8b**	5.80	58.74	1	1	0	1	4	419.32	6
**8c**	5.88	58.74	1	1	0	1	4	463.78	6
**9**	3.20	55.25	2	0	0	0	4	349.83	5
**10a**	4.51	64.71	2	0	0.05	1	4	397.88	6
**10b**	5.34	64.71	2	0	0	1	4	415.87	6
**10c**	5.55	64.71	1	0	0	1	4	433.86	6
**10d**	5.80	64.71	1	0	0.04	1	4	432.32	6
**10e**	5.88	64.71	1	0	0.02	1	4	476.77	6
**10f**	5.03	107.54	2	1	0.02	1	7	442.87	7
**12a**	5.63	51.21	1	0	0	0	4	394.87	4
**12b**	5.80	51.21	1	1	0	0	4	429.32	4
**12c**	6.38	51.21	1	1	0	0	4	473.77	4

^a^ Lipophilicity descriptor; ^b^ Polar surface area; ^c^ Solubility level. (0 = extremely low, 1 = very low but possible, 2 = low, 3 = good, 4 = optimal); ^d^ Absorption level. (0 = good, 1 = moderate, 2 = low, 3 = very low); ^e^ CYP2D6 Probability: 0–0.5 = non inhibitor; 0.5–1 = inhibitor.

## 3. Materials and Methods

### 3.1. Chemistry

Melting points were measured with a Stuart apparatus (Bibby Scientific Limited, Staffordshire, United Kingdom) and were uncorrected. The NMR spectra were recorded by Varian Gemini-300BB 300 MHz FT-NMR spectrometers (Varian Inc., Palo Alto, CA, USA). ^1^H and ^13^C spectra were run at 300 and 75 MHz, respectively, in deuterated dimethyl sulfoxide (DMSO-*d*_6_). Chemical shifts (δ_H_) are reported relative to TMS as internal standard. All coupling constant (*J*) values are given in hertz. Chemical shifts (δ_C_) are reported relative to DMSO-*d*_6_ as internal standards. The abbreviations used are as follows: s, singlet; d, doublet; m, multiplet. Microanalyses were carried out using Perkin Elmer PE 2400 CHN Elemental Analyzer (Akron, OH, USA) and the results were within ±0.4%. Analytical thin layer chromatography (TLC) on silica gel plates containing UV indicator was employed routinely to follow the course of reactions and to check the purity of products. All reagents and solvents were purified and dried by standard techniques.

#### 3.1.1. Synthesis of (1-(Aryl)-2-(2-(4-fluorophenyl)hydrazono)-2-(phenylsulfonyl)ethanones **6a**–**f**

To a solution of the appropriate amine **4a**–**f** (1 mL), hydrochloric acid (15 mL) was added. A solution of sodium nitrite (0.07 g, 1 mmol) in water (10 mL) was then gradually added with stirring. The resulting solution was added gradually within 30 min to a stirred cold solution (0–5 °C) of sulfone **3** (1 mmol) and sodium acetate trihydrate (0.26 g, 20 mmol) in ethanol (50 mL) and then left for 8 h in a refrigerator (4 °C). The resulting solid was collected by filtration, washed thoroughly with water, and dried to give the respective crude hydrazones which recrystallized from ethanol to give the corresponding hydrazones **6a**–**f**, respectively.

*1-(4-Chlorophenyl)-2-(2-phenylhydrazono)-2-(phenylsulfonyl)ethanone* (**6a**): Yellow powder (yield 71%), m.p. 170 °C; ^1^H-NMR (DMSO-*d*_6_) δ ppm: 7.01–7.26 (m, 2H, Ar-H), 7.57 (d, *J* = 8.1 Hz, 2H, Ar-H), 7.63–7.83 (m, 4H, Ar-H), 7.88 (d, *J* = 7.8 Hz, 2H, Ar-H), 7.95 (d, *J* = 8.1 Hz, 2H, Ar-H), 8.19 (d, *J* = 7.8 Hz, 2H, Ar-H), 11.46 and 12.31 (s, 1H, D_2_O exchangable, -NH); ^13^C-NMR (DMSO-*d*_6_) δ ppm: 114.37, 116.45, 127.68, 128.05, 129.34, 130.86, 131.65, 134.05, 137.27, 139.19, 139.76, 141.55, 142.43, 188.10; Anal. Calcd. for C_20_H_15_ClN_2_O_3_S (398.86): C, 60.22; H, 3.79; N, 7.02; Found C, 60.41; H, 3.70; N, 7.32.

*1-(4-Chlorophenyl)-2-(2-(4-flurophenyl)hydrazono)-2-(phenylsulfonyl)ethanone* (**6b**): Yellow powder (yield 74%); m.p. 181 °C, ^1^H-NMR (DMSO-*d*_6_) δ ppm: 7.11 (d, *J* = 8.7 Hz, 2H, Ar-H), 7.23–7.29 (m, 1H, Ar-H), 7.54 (d, *J* = 8.7 Hz, 2H, Ar-H), 7.67 (d, *J* = 7.5 Hz, 2H, Ar-H), 7.75 (dd, *J* = 7.5, 14.9, 2H, Ar-H), 7.89 (d, *J* = 8.1 Hz, 2H, Ar-H), 8.18 (d, *J* = 8.1 Hz, 2H, Ar-H), 11.49 and 12.33 (s, 1H, D_2_O exchangable, -NH); Anal. Calcd. for C_20_H_14_ClFN_2_O_3_S (416.85): C, 57.63; H, 3.39; N, 6.72; Found C, 57.94; H, 3.45; N, 6.52.

*1-(4-Chlorophenyl)-2-(2-(2,4-diflurophenyl)hydrazono)-2-(phenylsulfonyl)ethanone* (**6c**): Yellow powder (yield 80%), m.p. 177 °C; ^1^H-NMR (DMSO-*d*_6_) δ ppm: 7.08–7.28 (m, 3H, Ar-H), 7.53 (d, *J* = 8.7 Hz, 2H, Ar-H), 7.66 (d, *J* = 8.1 Hz, 2H, Ar-H), 7.77 (s, 1H, Ar-H), 7.79 (d, *J* = 8.4 Hz, 1H, Ar-H), 7.90 (d, *J* = 7.5 Hz, 1H, Ar-H), 8.16 (d, *J* = 7.5 Hz, 2H, Ar-H), 11.46 and 12.33 (s, 1H, D_2_O exchangable, -NH); ^13^C-NMR (DMSO-*d*_6_) δ ppm: 117.84, 118.00, 125.07, 128.06, 129.28, 130.85, 131.70, 134.69, 135.14, 137.54, 138.75, 139.49, 139.73, 184.91; Anal. Calcd. for C_20_H_13_ClF_2_N_2_O_3_S (434.84): C, 55.24; H, 3.01; N, 6.44; Found C, 55.02; H, 2.83; N, 6.59.

*1-(4-Chlorophenyl)-2-(2-(4-chlorophenyl)hydrazono)-2-(phenylsulfonyl)ethanone* (**6d**): Yellow powder (yield 85%), m.p. 213 °C; ^1^H-NMR (DMSO-*d*_6_) δ ppm: 7.07 (d, *J* = 8.7 Hz, 2H, Ar-H), 7.33 (m, 1H, Ar-H), 7.45 (d, *J* = 8.7 Hz, 2H, Ar-H), 7.68 (d, *J* = 6.3 Hz, 2H, Ar-H), 7.75 (dd, *J* = 7.5, 6.3 Hz, 2H, Ar-H), 7.90 (d, *J* = 7.5 Hz, 2H, Ar-H), 8.19 (d, *J* = 7.5 Hz, 2H, Ar-H), 11.47 and 12.31 (s, 1H, D_2_O exchangable, -NH); Anal. Calcd. for C_20_H_14_Cl_2_N_2_O_3_S (433.30): C, 55.44; H, 3.26; N, 6.47; Found C, 55.63; H, 3.39; N, 6.31.

*1-(4-Chlorophenyl)-2-(2-(4-bromophenyl)hydrazono)-2-(phenylsulfonyl)ethanone* (**6e**): Yellow powder (yield 87%), m.p. 232 °C; ^1^H-NMR (DMSO-*d*_6_) δ ppm: 7.01 (d, *J* = 8.7 Hz, 2H, Ar-H), 7.30 (d, *J* = 8.7 Hz, 2H, Ar-H), 7.54 (m, 1H, Ar-H), 7.68 (d, *J* = 7.8 Hz, 2H, Ar-H), ), 7.75 (dd, *J* = 7.8, 6.3 Hz, 2H, Ar-H), 7.90 (d, *J* = 7.5 Hz, 2H, Ar-H), 8.19 (d, *J* = 7.5 Hz, 2H, Ar-H), 11.47 and 12.30 (s, 1H, D_2_O exchangable, -NH); ^13^C-NMR ^1^H-NMR (DMSO-*d*_6_) δ ppm: 114.9, 116.31, 118.33, 128.10, 129.58, 131.67, 132.14, 133.79, 135.35, 137.45, 139.819, 139.99, 141.04, 187.99; Anal. Calcd. for C_20_H_14_BrClN_2_O_3_S (477.76): C, 50.28; H, 2.95; N, 5.86; Found C, 50.43; H, 3.10; N, 5.93.

*1-(4-Chlorophenyl)-2-(2-(4-nitrophenyl)hydrazono)-2-(phenylsulfonyl)ethanone* (**6f**): Orange powder (yield 60%), m.p. 216 °C; ^1^H-NMR (DMSO-*d*_6_) δ ppm: 7.20 (d, *J* = 9.0 Hz, 2H, Ar-H), 7.54 (d, *J* = 9.0 Hz, 2H, Ar-H), 7.69–7.79 (m, 1H, Ar-H), 7.82 (d, *J* = 8.1 Hz, 2H, Ar-H), 7.93 (d, J = 8.1 Hz, 2H, Ar-H), 8.16 (d, *J* = 9.0 Hz, 2H, Ar-H), 8.24–8.29 (m, 1H, Ar-H), 11.77 and 12.44 (s, 1H, D_2_O exchangable, -NH); Anal. Calcd. for C_20_H_14_ClN_3_O_5_S (443.86): C, 54.12; H, 3.18; N, 9.47; Found C, 54.29; H, 3.36; N, 9.64.

#### 3.1.2. Synthesis of 1-(Aryl)-2-(1-(4-chlorophenyl)-2-(phenylsulfonyl)ethylidene)hydrazines **8a**–**c**

To a stirred solution of the corresponding phenyl hydrazide **7a**–**c** (5 mmol) in absolute ethanol (20 mL), the sulfone derivative **3** (5 mmol) and catalytic amount of glacial acetic acid were added. The reaction mixture was heated under reflux for 6 h. The precipitate formed was collected by filtration while hot, washed with hot ethanol, dried and crystallized from ethanol/DMF to afford compounds **8a**–**c**.

*1-(Phenyl)-2-(1-(4-chlorophenyl)-2-(phenylsulfonyl)ethylidene)hydrazine* (**8a**): [25].

*1-(4-Chlorophenyl)-2-(1-(4-chlorophenyl)-2-(phenylsulfonyl)ethylidene)hydrazine* (**8b**): Yellow powder (yield 85%), m.p. 205 °C; ^1^H-NMR (DMSO-*d*_6_) δ ppm: 5.14 (s, 2H, -CH2), 7.09 (d, *J* = 9.0 Hz, 2H, Ar-H), 7.25 (d, *J* = 9.0 Hz, 2H, Ar-H), 7.30 (d, *J* = 8.7 Hz, 2H, Ar-H), 7.48 (t, *J* = 7.5, 1H, Ar-H), 7.57–7.65 (m, 2H, Ar-H), 7.69 (d, *J* = 8.7 Hz, 2H, Ar-H), 7.84 (d, *J* = 7.5 Hz, 2H, Ar-H), 9.86 (s, 1H, D_2_O exchangable, -NH); Anal. Calcd. for C_20_H_16_Cl_2_N_2_O_2_S (419.32): C, 57.29; H, 3.85; N, 6.68; Found C, 57.50; H, 4.04; N, 6.83.

*1-(4-Bromophenyl)-2-(1-(4-chlorophenyl)-2-(phenylsulfonyl)ethylidene)hydrazine* (**8c**): Yellow powder (yield 88%), m.p. 216 °C; ^1^H-NMR (DMSO-*d*_6_) δ ppm: 5.15 (s, 2H, -CH_2_), 7.04 (d, *J* = 8.7 Hz, 2H, Ar-H), 7.30 (d, *J* = 8.7 Hz, 2H, Ar-H), 7.37 (d, *J* = 8.7 Hz, 2H, Ar-H), 7.48 (t, *J* = 7.2, 1H, Ar-H), 7.57 (m, 2H, Ar-H), 7.69 (d, *J* = 8.7 Hz, 2H, Ar-H), 7.83 (d, *J* = 7.2 Hz, 2H, Ar-H), 9.87 (s, 1H, D_2_O exchangable, -NH); ^13^C-NMR (DMSO-*d*_6_) δ ppm: 70.30, 111.04, 114.97, 127.34, 137.47, 128.07, 129.26, 130.47, 130.72, 131.73, 132.15, 136.21, 139.12, 134.77; Anal. Calcd. for C_20_H_16_BrClN_2_O_2_S (463.77): C, 51.80; H, 3.48; N, 6.04; Found C, 52.06; H, 3.71; N, 6.26.

#### 3.1.3. Synthesis 1-(4-Chlorophenyl)-3-(dimethylamino)-2-(phenylsulfonyl)prop-2-en-1-one (**9**)

A mixture of sulfone **3** (10 mmol) and dimethylformamide-dimethylacetal (DMF-DMA) (12 mmol) was refluxed for 4 h. The residue was triturated with ether and the resulting yellow needles were collected by filtration, washed thoroughly with ether, dried and finally recrystallized from ethanol to afford the corresponding enaminone **9**. yellow powder (yield 75%), m.p. 190 °C; ^1^H-NMR (DMSO-*d*_6_) δ ppm: 3.09 (s, 6H, N(CH_3_)_2_), 7.46–7.63 (m, 7H, Ar-H), 7.73 (d, *J* = 7.2 Hz, 2H, Ar-H), 8.05 (s, 1H, = CH-); ^13^C-NMR (DMSO-*d*_6_) δ ppm: 41.73, 105.49, 126.52, 128.63, 130.14, 131.86, 125.15, 136.89, 128.99, 144.24, 155.19, 187.67; Anal. Calcd. for C_17_H_16_ClNO_3_S (349.83): C, 58.37; H, 4.61; N, 4.00; Found C, 58.60; H, 4.75; N, 4.15.

#### 3.1.4. Synthesis of 3-(Aryl amino)-1-(4-chlorophenyl)-2-(phenylsulfonyl)prop-2-en-1-one **10a**–**f**

A mixture of enaminone **9** (5 mmol) and the appropriate aniline **4a**–**f** (5 mmol) in glacial acetic acid (25 mL) was refluxed for 2 h, then left to cool. The solid product was filtered off, washed with water, dried and finally recrystallized from ethanol to afford the corresponding products **10a**–**f**, respectively.

*1-(4-Chlorophenyl)-3-(phenylamino)-2-(phenylsulfonyl)prop-2-en-1-one* (**10a**): White powder (yield 77%), m.p. 210 °C; ^1^H-NMR (DMSO-*d*_6_) δ ppm: 7.29–7.52 (m, 8H, Ar-H), 7.58 (d, *J* = 6.9 Hz, 2H, Ar-H), 7.65 (d, *J* = 6.9 Hz, 2H, Ar-H), 8.08 (d, *J* = 7.5 Hz, 2H, Ar-H), 8.00 and 8.55 (d, 1H, = CH-), 10.45 and 11.11 (s, 1H, D_2_O exchangable, -NH); ^13^C-NMR (DMSO-*d*_6_) δ ppm:109.65, 111.58, 118.53, 125.34, 126.98, 128.67, 130.64, 132.42, 135.87, 137.36, 139.20, 142.64, 149.83, 151.03, 187.89; Anal. Calcd. for C_21_H_16_ClNO_3_S (397.87): C, 63.39; H, 4.05; N, 3.52; Found C, 63.60; H, 4.23; N, 3.69.

*1-(4-Chlorophenyl)-3-((4-flurophenyl)amino)-2-(phenylsulfonyl)prop-2-en-1-one* **(10b)**: White powder (yield 78%), m.p. 219 °C; ^1^H-NMR (DMSO-*d*_6_) δ ppm: 7.22–7.38 (m, 4H, Ar-H), 7.57 (d, *J* = 9.3 Hz, 2H, Ar-H), 7.63 (d, *J* = 6.6 Hz, 2H, Ar-H), 7.71–7.76 (m, 1H, Ar-H), 7.88 (d, J = 8.7 Hz, 2H, Ar-H), 7.95 (d, *J* = 8.7 Hz, 2H, Ar-H), 8.07 and 8.47 (d, 1H, = CH-), 10.46 and 11.11 (s, 1H, D_2_O exchangable, -NH); Anal. Calcd. for C_21_H_15_ClFNO_3_S (415.87): C, 60.65; H, 3.64; N, 3.37; Found C, 60.86; H, 3.93; N, 3.20.

*1-(4-Chlorophenyl)-3-((2,4-diflurophenyl)amino)-2-(phenylsulfonyl)prop-2-en-1-one* (**10c**): White powder (yield 73%), m.p. 213 °C; ^1^H-NMR (DMSO-*d*_6_) δ ppm: 7.21 (d, *J* = 8.4 Hz, 1H, Ar-H), 7.34 (d, *J* = 8.7 Hz, 2H, Ar-H), 7.75 (s, 1H, Ar-H), 7.57 (d, *J* = 9.9 Hz, 2H, Ar-H), 7.63 (d, *J* = 9.9 Hz, 2H, Ar-H), 7.65–7.76 (m, 2H, Ar-H), 7.88 (d, *J* = 8.4 Hz, 1H, Ar-H), 7.95–8.02 (m, 1H, Ar-H), 8.06 and 8.54 (d, 1H, = CH-), 10.40 and 11.33 (s, 1H, D_2_O exchangable, -NH); ^13^C-NMR (DMSO-*d*_6_) δ ppm: 110.27, 112.28, 127.12, 127.92, 128.72, 128.87, 130.86, 133.94, 134.36, 136.49, 137.07, 139.19, 142.41, 152.10, 188.09; Anal. Calcd. for C_21_H_14_ClF_2_NO_3_S (433.85): C, 58.14; H, 3.25; N, 3.23; Found C, 58.46; H, 3.42; N, 3.00.

*1-(4-Chlorophenyl)-3-((4-chlorophenyl)amino)-2-(phenylsulfonyl)prop-2-en-1-one* (**10d**): White powder (yield 80%), m.p. 225 °C; ^1^H-NMR (DMSO-*d*_6_) δ ppm: 7.36–7.51 (m, 8H, Ar-H), 7.57–7.70 (m, 3H, Ar-H), 7.94–8.00 (m, 2H, Ar-H), 7.88 and 8.48 (d, 1H, = CH-), 10.46 and 10.93 (s, 1H, D_2_O exchangable, -NH); ^13^C-NMR (DMSO-*d*_6_) δ ppm: 111.96, 120.45, 127.24, 127.95, 128.67, 128.87, 129.20, 130.33, 132.58, 136.14, 137.38, 138.52, 142.81, 149.26, 189.78; Anal. Calcd. for C_21_H_15_Cl_2_NO_3_S (432.32): C, 58.34; H, 3.50; N, 3.24; Found C, 3.26; H, 3.75; N, 3.39.

*1-(4-Chlorophenyl)-3-((4-bromophenyl)amino)-2-(phenylsulfonyl)prop-2-en-1-one* (**10e**): White powder (yield 82%), m.p. 235 °C; ^1^H-NMR (DMSO-*d*_6_) δ ppm: 7.36–7.70 (m, 11H, Ar-H), 8.07 (d, *J* = 7.2 Hz, 2H, Ar-H), 7.95 and 8.47 (d, 1H, = CH-), 10.44 and 10.94 (s, 1H, D_2_O exchangable, -NH); Anal. Calcd. for C_21_H_14_BrClNO_3_S (476.77): C, 52.90; H, 3.17; N, 2.94; Found C, 53.12; H, 3.39; N, 3.17.

*1-(4-Chlorophenyl)-3-((4-nitrophenyl)amino)-2-(phenylsulfonyl)prop-2-en-1-one* (**10f**): Buff powder (yield 63%), m.p. 237 °C; ^1^H-NMR (DMSO-*d*_6_) δ ppm: 7.53–7.76 (m, 7H, Ar-H), 7.88–7.97 (m, 6H, Ar-H), 7.95 and 8.47 (d, 1H, = CH-), 10.46 and 10.96 (s, 1H, D_2_O exchangable, -NH); Anal. Calcd. for C_21_H_15_ClN_2_O_5_S (442.87): C, 56.95; H, 3.41; N, 6.33; Found C, 57.03; H, 3.72; N, 6.56.

#### 3.1.5. Synthesis of 1-(4-Aryl)-5-(4-chlorophenyl)-4-(phenylsulfonyl)-1*H*-pyrazole **12a**–**c**

A mixture of enaminone **9** (5 mmol) and the appropriate phenyl hydrazine **7a**–**c** (5 mmol) in absolute ethanol (30 mL) was refluxed for 6 h, then left to cool. The precipitated product was collected by filtration, washed with ethanol and dried. Recrystallization from ethanol afforded pyrazole derivatives **12a**–**c**, respectively.

*5-(4-Chlorophenyl)-1-phenyl-4-(phenylsulfonyl)-1H-pyrazole* (**12a**): White powder (yield 70%), m.p. 215 °C; ^1^H-NMR (DMSO-*d*_6_) δ ppm: 7.09 (d, *J* = 8.7 Hz, 2H, Ar-H), 7.17 (d, *J* = 8.4 Hz, 2H, Ar-H), 7.21–7.32 (m, 3 H, Ar-H), 7.40 (d, *J* = 8.4 Hz, 2H, Ar-H), 7.51 (d, *J* = 7.2 Hz, 2H, Ar-H), 7.58–7.67 (m, 1H, Ar-H), 7.68 (d, *J* = 7.2 Hz, 2H, Ar-H), 8.34 (s, 1H, pyrazole-H); Anal. Calcd. for C_21_H_15_ClN_2_O_2_S (394.87): C, 63.87; H, 3.83; N, 7.09; Found C, 63.99; H, 4.03; N, 7.01.

*1,5-Bis-(4-chlorophenyl)-4-(phenylsulfonyl)-1H-pyrazole* (**12b**): White powder (yield 84%), m.p. 225 °C; ^1^H-NMR (DMSO-*d*_6_) δ ppm: 7.20 (d, *J* = 7.8 Hz, 2H, Ar-H), 7.29 (d, *J* = 7.8 Hz, 2H, Ar-H), 7.35–7.48 (m, 4H, Ar-H), 7.51 (d, *J* = 7.8 Hz, 2H, Ar-H), 7.60–7.76 (m, 3H, Ar-H), 8.36 (s, 1H, pyrazole-H); Anal. Calcd. for C_21_H_14_Cl_2_N_2_O_2_S (429.32): C, 58.75; H, 3.29; N, 6.53; Found C, 58.94; H, 3.46; N, 6.26.

*1-(4-Bromophenyl)-5-(4-chlorophenyl)-4-(phenylsulfonyl)-1H-pyrazole* (**12c**): White powder (yield 84%), m.p. 245 °C; ^1^H-NMR (DMSO-*d*_6_) δ ppm: 7.20 (d, *J* = 4.2 Hz, 2H, Ar-H), 7.23 (d, *J* = 4.2 Hz, 2H, Ar-H), 7.30–7.40 (m, 2H, Ar-H), 7.44 (d, *J* = 6.3 Hz, 2H, Ar-H), 7.47–7.72 (m, 5H, Ar-H), 8.37 (s, 1H, pyrazole-H); Anal. Calcd. for C_21_H_14_BrClN_2_O_2_S (473.77): C, 53.24; H, 2.98; N, 5.91; Found C, 53.40; H, 3.15; N, 6.15.

### 3.2. Biological Evaluation

#### 3.2.1. Antifungal Activity

All strains were provided from culture collection of the Regional Center for Mycology and Biotechnology (RCMB), Al-Azhar University, Cairo, Egypt. Antifungal activities were expressed as the diameter of inhibition zones; agar well diffusion method was used. Holes (1 cm diameter) were digger in the agar using sterile cork borer in sterile malt agar plates for fungi, which had previously been uniformly seeded with tested microorganisms. The holes were filled by fungal filtrates (100 µL). Plates were left in a cooled incubator at 4 °C for one hour for diffusion and then incubated at 28 °C for tested fungi. Inhibition zones developed due to active antimicrobial metabolites were measured after 48 h of incubation for fungi. Fluconazole was used as antifungal positive control. The experiment was performed in triplicate and the average zone of inhibition was calculated [27].

#### 3.2.2. *In Vitro* Cytotoxic Activity

PC-3 human prostate cancer cells was grown in RPMI-1640 and supplemented with 10% heat inactivated FBS, 50 units/mL of penicillin and 50 g/mL of streptomycin and maintained at 37 °C in a humidified atmosphere containing 5% CO_2_. The cells were maintained as “monolayer culture” by serial subculturing. Cytotoxicity was determined using SRB method as previously described by Skehan *et al.* [34,35]. Exponentially growing cells were collected using 0.25% Trypsin-EDTA and seeded in 96-well plates at 1000–2000 cells/well in DMEM supplemented medium. After 24 h, cells were incubated for 48 h with various concentrations of the tested compounds. Following 48 h of treatment, the cells will be fixed with 10% trichloroacetic acid for 1 h at 4 °C. Wells were stained for 10 min at room temperature with 0.4% SRB dissolved in 1% acetic acid. The plates were air dried for 24 h and the dye was solubilized with Tris-HCl for 5 min on a shaker at 1600 rpm. The optical density (OD) of each well was measured spectrophotometrically at 564 nm with an ELISA microplate reader (ChroMate-4300, FL, USA). The IC_50_ values were calculated according to the equation for Boltzman sigmoidal concentration-response curve using the nonlinear regression fitting models (Graph Pad, Prism Version 5, GraphPad Software, Inc., San Diego, CA, USA). The results reported are means of at least three separate experiments. Statistical differences were analyzed according to one way ANOVA test wherein the differences were considered to be significant at *p* < 0.05.

### 3.3. Docking

The molecular docking of the tested compounds was performed using Discovery Studio 4/CDOCKER protocol (Accelrys Software Inc.). The protein crystallographic structure of *Mycobacterium* *tuberculosis* (Mycobacterium P450 DM) and co-crystallized fluconazole (PDB code: 1EA1) was downloaded from the Protein Data Bank (PDB). The protein was prepared for docking process according to the standard protein preparation procedure integrated in Accelry’s discovery studio 4 and prepared by prepare protein protocol. Fluconazole, **6a**, **8a**, **10a** and **10b** were drawn as a database and prepared by prepare ligand protocol to generate 3D structure and refine using CHARMM force field with full potential. Docking simulations were run using CDOCKER protocol where a maximum bad orientations was 800 and orientation vdW energy threshold was 300. Simulated annealing simulation would be then carried out consisting of a heating phase 700 K with 2000 steps and a cooling phase back to 5000 steps. The binding energy was calculated as a score to rank the docking poses. The top 10 docking poses would be finally saved. Docking poses were ranked according to their –CDOCKER interaction energy, and the top poses were chosen for analysis of interactions for each compound.

## 4. Conclusions

In an extension of our ongoing efforts towards developing potent antifungal agents, we developed a methodology for the synthesis of four novel target sulfone series, **6a**–**f**, **8a**–**c**, **10a**–**f** and **12a**–**c**. All the newly prepared phenyl sulfones were screened against invasive fungi, namely, *Candida* species (fluconazole-resistant *Candida albicans*, *Candida albicans*, *Candida tropicalis*, *Candida parapsilosis*) and *Asperigillus* species (*Aspergillus fumigatus*, *Aspergillus niger)*, dermatophytic fungi, namely *Trichophytons mentagrophyte* and *Microsporum canis* and the non-dermatophytic fungi *Syncephalastrum racemosum.* Fluconazole was used as a reference drug for the antifungal screening. The active compounds **6a**, **8a**, **10a**, **10b**, **10e** and **12a** showed no remarkable cytotoxicity against PC-3 cancer cell line with a selectivity index >11.19. The results evidenced that the p-fluoro phenyl aniline derivative **10b** exhibited excellent activity against all the candida species and was five times more potent than fluconazole on *C. albicans,* *C. tropicalis* and *C. Parapsilosis*.

Overall, compounds **8a** and **10b** could be considered as good lead candidates for further optimization of new potent anticandidal agents. Moreover, the unsubstituted phenyl hydrazine **6a** and the substituted aniline **10a** elicited broad spectrum activity against most of the tested organisms. On the other hand, the *p*-bromo aniline derivative **10e** and the pyrazole derivative **12a** could also act as a starting point in the process of discovery of new anti-aspergillosis and anti-dermatophytic agents. A docking study of the most active derivatives **6a**, **8a**, **10a** and **12b** was performed on Cyp-P450 DM enzyme (PDB code: 1EA1) and presented a rationale for the activity of these compounds. Furthermore, a theoretical kinetic study was established to predict the ADME of the active derivatives. Finally, the work also described an X-ray study of the starting 1-(4-chlorophenyl)-2-(phenylsulfonyl)ethanone **3**.

## Supplementary Materials

Supplementary materials can be accessed at: http://www.mdpi.com/1420-3049/21/1/114/s1.
