# Supplementary material for: Synthesis, Biological Evaluation and Molecular Docking of Certain Sulfones as Potential Nonazole Antifungal Agents
Source: Molecules. 2016 Jan 20;21(1):114. doi: 10.3390/molecules21010114 (PMC6274496; doi:10.3390/molecules21010114)
Supplement: Supplementary file 1 [file molecules-21-00114-s001.pdf]

Supplementary Materials: Synthesis, Biological Evaluation and Molecular Docking of Certain Sulfones as Potential Nonazole Antifungal Agents

Mohamed Fares, Mohamed A. Said, Muhammad A. Alsherbiny, Radwa A. Eladwy, Hadia Almahli, Marwa M. Abdel-Aziz, Hazem A. Ghabbour, Wagdy M. Eldehna and Hatem A. Abdel-Aziz

X-ray crystallographic analysis: Crystal of compound **3** was obtained by slow evaporation from solution of ethanol/DMF. The measurements of the crystal were performed on a Bruker SMART APEX II D8 Venture diffractometer with graphite-monochromated Cu *Kα* radiation (λ = 1.54178 Å) at 293 K. The structure was solved by direct method and refined with SHELXTL. E-maps provided the positions of all the non-H-atoms. The full-matrix least-squares refinement was carried out on F^2's using anisotropic temperature factors for all non-*H*-atoms. Crystallographic data for the structure reported in this paper have been deposited at the Cambridge Crystallographic Data Center and allocated with the deposition number: CCDC 1430111.

Table S1. Crystallographic data and refinements for compound **3**.

**Crystal data**	
$C_{14}H_{11}ClO_3S$	V = 1292.27 (8) Å^3
M_r = 294.74	Z = 4
Monoclinic, $P2_1/c$	Cu *Kα* radiation
a = 5.7258 (2) Å	μ = 4.14 mm^{-1}
b = 9.1203 (4) Å	T = 293 K
c = 25.0426 (8) Å	0.50 × 0.11 × 0.07 mm
β = 98.824 (3)°	F(000) = 608
**Data collection**	
CCD area detector diffractometer	R_{int} = 0.104
7841 measured reflections	θ_{max} = 62.5°
2021 independent reflections	775 reflections with $I > 2\sigma(I)$
**Refinement**	
$R[F^2 > 2\sigma(F^2)]$ = 0.103	0 restraints
$wR(F^2)$ = 0.311	H atoms treated by a mixture of independent and constrained refinement
S = 0.92	$w = 1/[\sigma^2(F_o^2) + (0.1895P)^2]$ where $P = (F_o^2 + 2F_c^2)/3$
2021 reflections	$\Delta\rho_{max}$ = 0.49 e·Å^{-3}
173 parameters	$\Delta\rho_{min}$ = −0.58 e·Å^{-3}

The crystallographic structure of **3** is represented in Figure 2. The single crystal X-ray study on this derivative unambiguously defines the exact structure. Crystal packing of **3** which showed the intermolecular hydrogen bond C10—H10A···O2 is presented in Figure 3. The crystallographic data and refinement for the crystal was presented in Table S1. Selected geometric parameters of compound **3** presented in Table S2 (Supplementary Materials). Hydrogen-bond geometry of **3** is illustrated in Table S3.

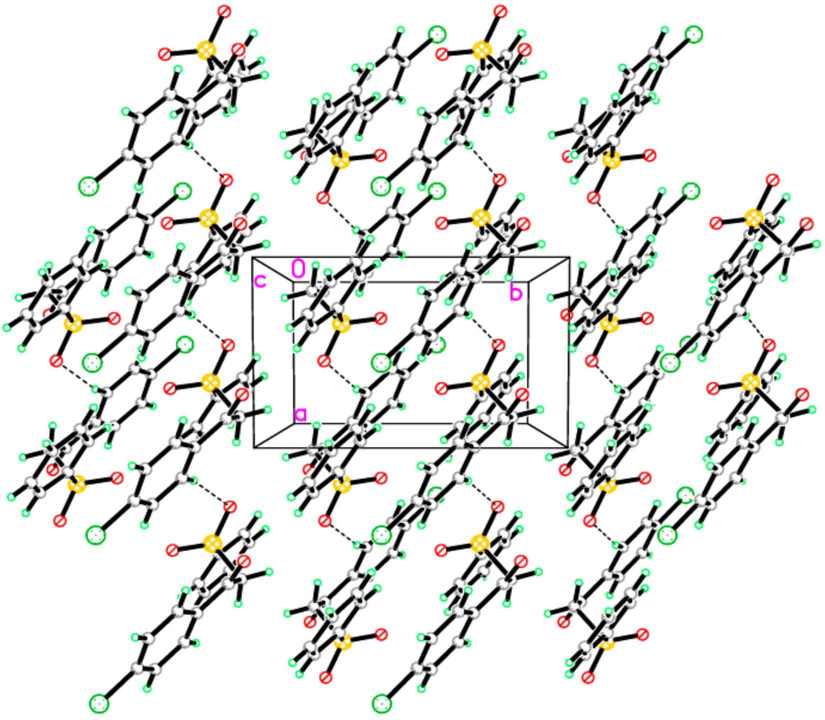

Figure S1. Crystal packing of **3** showing intermolecular hydrogen bonds as dashed lines.

Table S2. Selected geometric parameters (Å, °) for compound **3**.

**Bond distance**			
Cl1—C12	1.738 (9)	S1—C6	1.753 (8)
S1—O1	1.416 (5)	S1—C7	1.767 (8)
S1—O2	1.446 (7)	O3—C8	1.218 (11)
**Bond angle**			
O1—S1—O2	118.7 (3)	S1—C6—C5	120.5 (7)
O1—S1—C6	109.5 (3)	S1—C7—C8	110.3 (6)
O1—S1—C7	107.3 (3)	O3—C8—C7	117.4 (7)
O2—S1—C6	108.7 (4)	O3—C8—C9	122.7 (7)
O2—S1—C7	107.7 (3)	Cl1—C12—C11	119.9 (7)
C6—S1—C7	103.9 (4)	Cl1—C12—C13	118.6 (7)
S1—C6—C1	118.9 (6)		

Table S3. Hydrogen-bond geometry (Å, °) of **3**.

D—H···*A*	*D*—H	H···*A*	*D*···*A*	*D*—H···*A*
C10—H10A···O2 [i]	0.9300	2.5000	3.403 (10)	163.00

Symmetry codes: (i) *x* + 1, *y*, *z*.
